# Epidemiological characteristics of different patterns of obesity among adults in rural communities of south-east Nigeria: a population-based cross-sectional study

**DOI:** 10.1186/s40795-022-00552-5

**Published:** 2022-06-27

**Authors:** Rufina N. B. Ayogu, Mmesoma G. Ezeh, Elizabeth A. Udenta

**Affiliations:** Department of Nutrition and Dietetics, University of Nigeria, Nsukka, Nigeria

**Keywords:** Abdominal obesity, General obesity, Combined obesity, Predictors, Adults, Rural, South-East Nigeria

## Abstract

**Background:**

Obesity is a complex disease characterised by excess fat accumulation and health risks. There is paucity of data on epidemiology of obesity patterns among adults in rural Nigeria. This study aimed to provide current data on the prevalence and predictors of three patterns of obesity (abdominal obesity alone, general obesity alone and a combination of both) among adults in three rural communities of Enugu State, South-east Nigeria to enhance specific obesity prevention and control programmes/interventions.

**Methods:**

This population-based cross-sectional study involved 500 adults selected through a six-stage random sampling technique. Questionnaire was used to obtain data on socio-economic, dietary and lifestyle characteristics of the respondents. Weight, height and waist circumference were measured by standard procedures. Body mass index was used to assess general obesity while abdominal obesity was assessed through waist circumference. Each respondent was classified under only one of the three patterns: general obesity alone, abdominal obesity alone and combined obesity. Binary and multivariate logistic regression analyses were used to assess the predictors. Significance was set *P*<0.05.

**Results:**

Prevalence of abdominal obesity alone was 6.0%. General obesity alone was found among 31.4% and 45.6% were affected by combined obesity. Being a female (AOR:0.35, 95% C.I.: 0.14, 0.88) and not skipping meals (AOR:0.24, 95% C.I.: 0.10, 0.55) were associated with less likelihood of abdominal obesity but ≥3 times daily meal intake increased the risk by 2.52 (AOR:2.52, 95% C.I.:1.10, 5.75). Predictors of general obesity alone were age 41-60 years (AOR:1.84, 95% C.I.:1.14, 2.97), being a female (AOR:7.65, 95% C.I.:4.77, 12.26) and having any form of formal education (AOR:2.55, 95% C.I.:1.10, 5.91). Combined obesity was less likely among 41-60 year-olds (AOR:0.36, 95% C.I.:0.23, 0.56) and females (AOR:0.21, 95% C.I.:0.13, 0.32). Never married (AOR:1.94, 95% C.I.:1.03, 3.67) and vigorous physical activities (AOR:1.81, 95% C.I.:1.08, 3.02) increased the risk of combined obesity by almost 2.

**Conclusions:**

Prevalence of abdominal obesity alone, general obesity alone and combined obesity were high. They were functions of age, sex, never married, having any form of formal education, skipping meals, ≥3 daily meal intake and self-reported vigorous physical activity. Focused nutrition and health education are recommended strategies for prevention and control of obesity.

## Background

Obesity is a complex multifactorial disease because it is a consequence of interplay of numerous interconnected genetic and environmental factors with prolonged energy imbalance in which energy consumed exceeds energy expended leading to excess fat deposits in the body. In certain cultures, as seen in many African countries, overweight is associated with richness, health, strength and fertility [1]. In 2016, more than 1.9 billion (39%) adults aged 18 years and older were overweight globally; of these, over 650 million (13%) were obese [2]. It is projected that by 2030, the global prevalence of overweight and obesity will rise to 2.16 billion and 1.12 billion, respectively [3]. According to World Health Organization (WHO) [4], the prevalence of obesity has nearly doubled since 1980. In Nigeria, obesity has been reported among adults with prevalence ranging from 18.1 – 22.2% [5] and 64% [6] and higher prevalence among females and urban dwellers. This calls for regular evaluation of obesity prevalence and predictors to show trend and ensure effective prevention and control. This will save medical costs since 40% of Nigerians live below the nation’s poverty line of 137,430 Naira ($381.75) per year as at 2018 [7].

Currently, there is rapid upsurge in the prevalence of non-communicable chronic diseases (NCDs) with younger persons especially adolescents being affected. Obesity increases the likelihood of diabetes, hypertension, coronary heart disease, stroke and certain types of cancer [4]. Cardiovascular diseases (mainly heart disease and stroke which were leading causes of death in 2012), diabetes, musculoskeletal disorders (especially osteoarthritis) and some cancers are common health consequences of overweight and obesity [2]. Prognosis of diabetes and cardiovascular diseases (CVDs) are exacerbated by overweight and obesity and being overweight or obese contributes significantly to multiple morbidity and high mortality rate in various countries around the world [5]. In Africa, deaths from NCDs are rising faster than anywhere else in the world as a result of a global epidemic of smoking, unhealthy diet, harmful use of alcohol and physical inactivity with explosion in obesity and high blood pressure rates [8].

A study conducted in Alameda, California [9] linked obesity with depression and reported that obesity at baseline was associated with increased risk of depression 5 years later even after controlling for depression and an array of covariates at baseline. In a population-based cohort study of 5.24 million UK adults that assessed the association between body mass index (BMI) and the risk of 22 specific cancers, Bhaskaran et al. [10] reported that elevated BMI was associated with 17 out of 22 cancers; each 5 kg/m^2^ increase in BMI was roughly linearly associated with cancers of the uterus, gall bladder, kidney, cervix, thyroid, and leukaemia with positive associations with liver, colon, ovarian and postmenopausal breast cancers. According to Kearns et al. [11], prevalence of chronic diseases generally increased with increasing BMI and one-unit decrease in BMI results in 26 and 28 fewer cases of chronic diseases per 1,000 men and women, respectively.

Overweight and obesity result from interplay of genetic, nutritional, environmental and socio-economic variables. According to International Diabetes Federation [12], there is an overwhelming moral, medical and economic imperative to identify individuals with obesity early since obesity is key to metabolic syndrome which is driving the twin global epidemics of type 2 diabetes and CVDs. Besides, few studies have examined the association of socioeconomic, lifestyle and dietary characteristics of adults with abdominal obesity alone, general obesity alone and combined obesity concurrently. This study aimed at assessing rural adult population (as about 48% of Nigerians live in rural areas) in Udenu local government area (LGA), Enugu North senatorial zone of Enugu State, South-east Nigeria for the prevalence and predictors of three patterns of obesity (abdominal obesity alone, general obesity alone and a combination of abdominal and general obesity). This preliminary study will trigger lifestyle modification interventions to enhance a holistic approach aimed at obesity prevention, control or reversal and prevent the development of diabetes, cardiovascular diseases and cancers.

## Methods

### Area of study

The study was conducted in three rural communities (Obollo-afor, Ezimo-Ulo and Orba) in Udenu LGA, Enugu North senatorial zone of Enugu state, South-east Nigeria. The LGA is subdivided into three development centres with a population of about 255,889 as at 2018 based on annual projected increase of 3.0%. The inhabitants are mainly farmers, traders and civil servants. Foods commonly consumed in the area are cocoyam, bambara nut, yam, rice, cassava, maize, and cowpea of different species.

### Study design and participants

A cross sectional survey design was used to assess the prevalence and predictors of abdominal obesity alone, general obesity alone and combined obesity among 500 free living adults aged 20 – 60 years in three communities of Udenu LGA of Enugu state. Participants who were sick, pregnant or lactating at the time of data collection were excluded from the study.

### Sample size determination

A single proportion Cochran’s formula $n=\frac{t^2\times p\left(1-p\right)}{m^2}$with p value of 20%, 5% margin of error and 5% non-response rate was used to obtain a sample size of 500. This figure was distributed among the three communities using probability proportional to size: 40% (200) for Obollo-afor, 30% (150) for Ezimo-Ulo and 30% (150) for Orba.

### Sampling technique

Multistage sampling technique was used in sample selection. The first stage involved selection of one community (town) from each development centre through simple random sampling by balloting without replacement giving a total of three communities in all. Udenu LGA has three development centres. In the second stage, two villages were selected from each community using simple random sampling technique by balloting without replacement. In the third stage, clans (*umunna*) in the villages were listed alphabetically and selected using simple random sampling by balloting without replacement. The fourth stage involved selection of living houses from the clans. Houses in the clans were numbered and selected using systematic random sampling; one out of every 10 houses was selected. Enumeration of houses was made possible by village and clan heads. A total of 150, 150 and 200 houses were selected from Ezimo-ulo, Orba and Obollo Afor, respectively. Probability proportional to size (PPS) was used to assign these numbers. In the fifth stage, households were selected from the houses (one per house) using simple random sampling technique by balloting without replacement. The sixth stage involved selection of only one adult (20–60 years) from each selected household through simple random sampling by balloting without replacement. This has been described elsewhere [13].

### Ethical clearance and consent to participate

Ethical approval was obtained from the Health Research Ethics Committee of the University of Nigeria Teaching Hospital, Ituku-Ozalla, Enugu State (NHREC/05/01/2008B-FWA00002458-1RB00002323). Informed consent was obtained verbally from each respondent after detailed explanation of what the study entailed.

#### Data collection methods

##### Questionnaire

Socio-economic, lifestyle and dietary characteristics of the respondents were elicited through an interviewer-administered questionnaire. Self-reported physical activity level was assessed with the WHO Global physical activity questionnaire. Respondents were requested to recall types, frequency and duration (in minutes) of all activities engaged in within the week preceding data collection. Total physical activity was calculated by summing all the minutes spent on each physical activity category to determine if they attained the prescribed WHO weekly recommendation of at least 150 minutes of moderate intensity or 75 minutes of vigorous intensity. An individual was classified as physically active if he/she achieved this minimum recommendation [14]. Data on dietary diversity score was obtained through 24-hour dietary recall based on 9 food groups as described by Ayogu [15]. Any food group consumed during the 24-hour period was scored one. The scores were summed up for each participant to obtain his/her dietary diversity score. The scores were categorised as low (≤3), medium (4-5) and high (≥6) dietary diversity score.

##### Anthropometry

Participants were weighed to the nearest 0.1 kg using a Hanson’s weighing scale (capacity of 120 kg); height was measured to the nearest 0.1 cm using a metre rule attached to a wooden pole. The procedures have been described elsewhere [16]. Weight and height were taken twice and the mean value was used in statistical analysis provided the difference between the two weight measurements did not exceed 0.2 kg and height did not exceed 0.5 cm. If the measurements exceeded 0.2 kg for weight and 0.5 cm for height, a third measurement was taken and the mean of the closest two measurements (with difference ≤ 0.2 kg or 0.5 cm) were used in statistical analysis [17]. Body mass index (ratio of weight in kg to height in metres squared) was calculated and BMI of ≥ 30 Kg/m^2^ was taken as general obesity. Waist circumference (WC) was measured with a flexible non-stretch tape placed on the midpoint between the top of the iliac crest and the bottom of the rib cage where the last palpable rib is found. The tape was held firm without compressing the tissues and the reading was taken to the nearest 0.1 cm at the end of normal expiration. Values ≥ 94 cm for males and 80 cm for females were used to determine the prevalence of abdominal adiposity [12]. Presence of both general and abdominal obesity in an individual was described as combined obesity. Obese participants were classified into one of the three patterns of obesity: abdominal obesity alone, general obesity alone and combined obesity.

##### Statistical analysis

Coded data were analysed through IBM Statistical Product and Service Solutions (SPSS) version 21.0. The results of descriptive statistical analysis were presented as frequencies and percentages. Binary and multivariate logistic regression analyses were used to assess associations between variables. Crude and adjusted odds ratios were reported. Results with 95% precision (*P*<0.05) were taken as significant.

## Results

Table 1 shows univariate analysis of the general characteristics of the respondents. The study involved 52.0% of adults aged 20 – 40 years and 48.0% of those aged 41 – 60 years. There were more females (57.4%) than males (42.6%); more (93.6%) low monthly income earners of less than 50,000 Naira and more (83.2%) ever married adults. Only 8.0% had no formal education. Those that consumed alcohol within 30 days and 12 months preceding the study were 40.6 and 56.6%, respectively. Whereas 60.6% skipped meals, 55.4% consumed meals ≥3 times daily, 79.0 and 94.6% reported physical activity of lighted/moderate intensity and good health status, respectively. Majority (74.4%) had high dietary diversity scores.

**Table 1:** Univariate analysis of general characteristics of the respondents

Variables	Frequency ***N***=500	Percent
**Age (years)**		
20-40	260	52.0
41-60	240	48.0
**Sex**		
Female	287	57.4
Male	213	42.6
**Monthly income**		
<50,000 Naira	468	93.6
≥50,000 Naira	32	6.4
**Marital status**		
Ever married	416	83.2
Never married	84	16.8
**Education**		
Had any form of formal education	460	92.0
No formal education	40	8.0
**Alcohol consumption within 30 days preceding the study**		
Yes	203	40.6
No	297	59.4
**Alcohol consumption within 12 months preceding the study**		
Yes	283	56.6
No	217	43.4
**Ever skipped meals**		
Yes	303	60.6
No	197	39.4
**Daily meal intake**		
<3 times	223	44.6
≥3 times	277	55.4
**Self-reported physical activity**		
Light/moderate intensity	395	79.0
Vigorous intensity	105	21.0
**Dietary diversity score**		
High	372	74.4
Low/medium	128	25.6
**Self-reported health status**		
Good	473	94.6
Poor	27	5.4
**Dietary diversity score**		
Low/medium	128	25.6
High	372	74.4

One USD is equivalent to 410.84 Naira

Table 2 illustrates the categories of body mass index and patterns of obesity among the respondents. The 20–40 year-olds had higher prevalence of underweight (100.0%) and overweight (61.8%). Prevalence of obesity class I (54.0%), class II (75.6%) and class III (75.0%) were higher among those aged 41-60 years. Prevalence of underweight (75.0%), obesity class II (86.7%), and class III (81.8%) were higher among females; males were affected more by overweight (52.2%) and obesity class I (51.1%). These differences were significant (*P*<0.001). Only 17.0% of the respondents did not have any form of obesity; 6.0% had abdominal obesity alone, 31.4% had general obesity alone and 45.6% were affected by combined obesity. Abdominal obesity alone had higher prevalence among 20-40 year-olds (53.3%) and females (76.7%). Prevalence of general obesity alone was higher among those aged 20-40 years (64.3%) and males (74.5%). Those aged 41-60 years (63.6%) and females (77.6%) had higher prevalence of combined obesity than their counterparts. These relationships were also significant (*P*<0.001).

**Table 2:** Categories of body mass index and patterns of obesity among the respondents

	Age (years)		Sex		
	20-40	41-60	Female	Male	
Variables	N(%)	N(%)	N(%)	N(%)	Total
**Body mass index categories (**kg/m^2^)					
Underweight (<18.5)	4(100.0)	0(0.0)	3(75.0)	1(25.0)	4(0.8)
Normal (18.5-24.99)	73(65.8)	38(34.2)	66(59.5)	45(40.5)	111(22.2)
Overweight (25.0-29.99)	97(61.8)	60(38.2)	75(47.8)	82(52.2)	157(31.4)
Obesity class I (30-34.99)	64(46.0)	75(54.0)	68(48.9)	71(51.1)	139(27.8)
Obesity class II (35-39.99)	11(24.4)	34(75.6)	39(86.7)	6(13.3)	45(9.0)
Obesity class III (≥40)	11(25.0)	33(75.0)	36(81.8)	8(18.2)	44(8.8)
P	<0.001		<0.001		
**Patterns of obesity**					
None	60(70.6)	25(29.4)	47(55.3)	38(44.7)	85(17.0)
Abdominal obesity alone	16(53.3)	14(46.7)	23(76.7)	7(2.3)	30(6.0)
General obesity alone	101(64.3)	56(35.7)	40(25.5)	117(74.5)	157(31.4)
^a^Combined obesity	83(36.4)	145(63.6)	177(77.6)	51(22.4)	228(45.6)
P	<0.001		<0.001		
**Total**	**260(100.0)**	**240(100.0)**	**287(100.0)**	**213(100.0)**	**500(100.0)**

*P* values were generated through Chi square analysis

^a^Having both general and abdominal obesity

Table 3 displays the predictors of abdominal obesity alone among the adults. Females were less likely to have abdominal obesity alone than males (AOR:0.35, 95% C.I.:0.14, 0.88, *P*<0.05) . Those who skipped meals had 76% higher probability of having abdominal obesity alone than those who did not skip meals (AOR:0.24, 95% C.I.: 0.10, 0.55; *P*<0.01). The risk of abdominal obesity was 3 times higher among those with ≥3 times daily meal intake (AOR:2.52, 95% C.I.:1.10, 5.75; *P*<0.05).

**Table 3:** Predictors of abdominal obesity alone among the adults

	Abdominal obesity alone					
Variables	Present^**a**^	Absent	COR(95% C.I.)	P	AOR(95% C.I.)	P
**Age (years)**						
20-40	16(6.2)	244(93.8)				
41-60	14(5.8)	226(94.2)	1.06(0.51-2.22)	0.880	1.36(0.60-3.11)	0.466
**Sex**						
Male	7(3.3)	206(96.7)				
Female	23(8.0)	264(92.0)	0.39(0.16-0.93)	<0.05	0.35(0.14-0.88)	<0.05
**Monthly income (Naira)**^**b**^						
< 50,000	29(6.2)	439(93.8)				
≥ 50,000	1(3.1)	31(96.9)	2.05(0.27-15.54)	0.488	2.76(0.35-22.04)	0.339
**Marital status**						
Ever married	26(6.3)	390(93.8)				
Never married	4(4.8)	80(95.2)	1.33(0.45-3.93)	0.602	1.28(0.39-4.22)	0.690
**Education**						
No formal education	0(0.0)	40(100.0)				
Had any form of formal education	30(6.5)	430(93.5)	NA			
**Alcohol consumption within 30 days preceding the study**						
Yes	6(3.0)	196(97.0)				
No	24(8.1)	274(91.9)	0.35(0.14-0.87)	<0.05	0.46(0.13-1.62)	0.226
**Alcohol consumption within 12 months days preceding the study**						
Yes	11(3.9)	272(98.1)				
No	19(8.8)	198(91.2)	0.42(0.20-0.91)	<0.05	0.72(0.25-2.07)	0.545
**Ever skipped meals**						
Yes	10(3.3)	293(96.7)				
No	20(10.2)	177(89.8)	0.30(0.14-0.66)	<0.01	0.24(0.10-0.55)	<0.01
**Daily meal intake**						
<3 times	16(7.2)	207(92.8)				
≥3 times	14(5.1)	263(94.9)	1.45(0.69-3.04)	0.323	2.52(1.10-5.75)	<0.05
**Self-reported physical activity**						
Light/moderate intensity	23(5.8)	372(94.2)				
Vigorous intensity	7(6.7)	98(93.3)	0.87(0.36-2.08)	0.746	0.76(0.30-1.91)	0.557
**Dietary diversity score**						
High (≥6 scores)	7(5.5)	121(94.5)				
Low/medium (<6 scores)	23(6.2)	349(93.8)	0.88(0.37-2.10)	0.769	1.12(0.45-2.80)	0.808
**Self-reported health status**						
Poor	0(0.0)	27(100.0)				
Good	30(6.3)	443(93.7)	NA			

*COR* Crude odds ratio, *AOR* Adjusted odds ratio, *NA* Not applicable

^a^6.0%

^b^One USD= 410.84 Naira

Table 4 illustrates predictors of general obesity alone among the respondents. General obesity alone was more likely among the 41–60 years (AOR:1.84. 95% C.I.: 1.14, 2.97; *P*<0.05), females (AOR:7.65, 95% C.I.:4.77, 12.26; *P*<0.001) and those who had any form of formal education (AOR:2.55, 95% C.I.: 1.10, 5.91; *P*<0.05).

**Table 4:** Predictors of general obesity alone among the respondents

	General obesity alone					
Variables	Present^**a**^	Absent	COR(95% C.I.)	P	AOR(95% C.I.)	P
**Age (years)**						
20-40	101(38.8)	159(61.2)				
41-60	56(23.3)	184(76.7)	2.09(1.41-3.08)	<0.001	1.84(1.14-2.97)	<0.05
**Sex**						
Male	116(54.5)	97(45.5)				
Female	41(14.3)	246(85.7)	7.18(4.68-10.99)	<0.001	7.65(4.77-12.26)	<0.001
**Monthly income (Naira)**^**b**^						
< 50,000	144(30.8)	324(69.2)				
≥ 50,000	13(40.6)	19(59.4)	0.65(0.31-1.35)	0.248	0.47(0.20-1.11)	0.084
**Marital status**						
Ever married	121(29.1)	295(70.9)				
Never married	36(42.9)	48(57.1)	0.55(0.34-0.89)	<0.05	0.89(0.49-1.60)	0.697
**Education**						
No formal education	12(30.0)	28(70.0)				
Had any form of formal education	145(31.5)	315(68.5)	0.93(0.46-1.88)	0.842	2.55(1.10-5.91)	<0.05
**Alcohol consumption within 30 days preceding the study**						
Yes	78(38.6)	124(61.4)				
No	79(26.5)	219(73.5)	1.74(1.19-2.56)	<0.01	1.85(0.98-3.50)	0.059
**Alcohol consumption within 12 months days preceding the study**						
Yes	98(34.6)	185(65.4)				
No	59(27.2)	158(72.8)	1.42(0.96-2.09)	0.076	0.67(0.35-1.30)	0.238
**Ever skipped meals**						
Yes	99(32.7)	204(67.3)				
No	58(29.4)	139(70.6)	1.16(0.79-1.72)	0.447	1.07(0.68-1.68)	0.782
**Daily meal intake**						
<3 times	78(35.0)	145(65.0)				
≥3 times	79(28.5)	198(71.5)	1.35(0.92-1.97)	0.122	1.16(0.74-1.80)	0.523
**Self-reported physical activity**						
Light/moderate intensity	118(29.9)	277(70.1)				
Vigorous intensity	39(37.1)	66(62.9)	0.72(0.46-1.13)	0.155	0.86(0.51-1.45)	0.575
**Dietary diversity score**						
High (≥6 scores)	41(32.0)	87(68.0)				
Low/medium (<6 scores)	116(31.2)	256(68.8)	1.04(0.68-1.60)	0.858	0.80(0.49-1.31)	0.376
**Self-reported health status**						
Poor	11(40.7)	16(59.3)				
Good	146(30.9)	327(69.1)	1.54(0.70-3.40)	0.285	1.21(0.49-3.02)	0.677

*COR* Crude odds ratio, *AOR* Adjusted odds ratio

^a^31.4%

^b^One USD=410.84 Naira

Predictors of combined obesity among the adults are presented in Table 5. Older adults of 41 – 60 years had 36% higher odds of having combined obesity (AOR: 0.36, 95% C.I.: 0.23, 0.56; *P*<0.001). There is 79% likelihood of combined obesity among males than females (AOR: 0.21, 95% C.I.: 0.13, 0.32; *P*<0.001). Never married (AOR: 1.94, 95% C.I.: 1.03, 3.67; *P*<0.05) and self-reported vigorous physical activity (AOR: 1.81, 95% C.I.:1.08, 3.02; *P*<0.05) were associated with almost 2 times lower odds of combined obesity.

**Table 5:** Predictors of combined obesity among the adults

	Combined obesity					
Variables	Present^**a**^	Absent	COR(95% C.I.)	P	AOR(95% C.I.)	P
**Age (years)**						
20-40	83(31.9)	177(68.1)				
41-60	145(60.4)	95(39.6)	0.31(0.21-0.44)	<0.001	0.36(0.23-0.56)	<0.001
**Gender**						
Male	51(23.9)	162(76.1)				
Female	177(61.7)	110(38.3)	0.20(0.13-0.29)	<0.001	0.21(0.13-0.32)	<0.001
**Monthly income (Naira)**^**b**^						
< 50,000	212(45.3)	256(54.7)				
≥ 50,000	16(50.0)	16(50.0)	0.83(0.41-1.70)	0.606	0.83(0.38-1.85)	0.653
**Marital status**						
Ever married	210(50.5)	206(49.5)				
Never married	18(21.4)	66(78.6)	3.74(2.15-6.51)	<0.001	1.94(1.03-3.67)	<0.05
**Education**						
No formal education	27(67.5)	13(32.5)				
Had any form of formal education	201(43.7)	259(56.3)	2.68(1.35-5.32)	<0.01	1.25(0.56-2.78)	0.581
**Alcohol consumption within 30 days preceding the study**						
Yes	95(47.0)	107(53.0)				
No	133(44.6)	165(55.4)	1.10(0.77-1.58)	0.597	1.01(0.55-1.82)	0.987
**Alcohol consumption within 12 months days preceding the study**						
Yes	132(46.6)	151(53.4)				
No	96(44.2(	121(55.8)	1.10(0.77-1.57)	0.593	1.66(0.93-3.00)	0.089
**Ever skipped meals**						
Yes	135(44.6)	168(55.4)				
No	93(47.2)	104(52.8)	0.90(0.63-1.29)	0.561	0.99(0.65-1.52)	0.962
**Daily meal intake**						
<3 times	93(41.7)	130(58.3)				
≥3 times	135(48.7)	142(51.3)	0.75(0.53-1.07)	0.117	0.83(0.55-1.27)	0.393
**Self-reported physical activity**						
Light/moderate intensity	189(47.8)	206(52.2)				
Vigorous intensity	39(37.1)	66(62.9)	1.55(1.00-2.42)	0.051	1.81(1.08-3.02)	<0.05
**Dietary diversity score**						
High (≥6 scores)	54(42.2)	74(57.8)				
Low/medium (<6 scores)	174(46.8)	198(53.2)	0.83(0.55-1.25)	0.369	0.91(0.57-1.45)	0.691
**Self-reported health status**						
Poor	14(51.9)	13(48.1)				
Good	214(45.2)	259(54.8)	1.30(0.60-2.83)	0.504	2.10(0.82-5.36)	0.122

*COR* Crude odds ratio, *AOR* Adjusted odds ratio

^a^45.6%

^b^One USD=410.84 Naira

## Discussion

This cross sectional survey is part of a larger project carried out between June and August, 2018.

Only 17.0% of the adults were found without any form of obesity with a little less than half having combined (both abdominal and general) obesity. These findings are in line with the findings of other researchers [5, 6, 18, 19]. In a study on northern Nigerian adult population, Wahab et al. [19] reported general obesity prevalence of 21.0% with a higher prevalence among females contrary to the findings of this study. Our observation was similar to Kearns et al.’s [10] report of a higher prevalence of general obesity among males (16.1%) than females (13.4%). According to Chukwuonye et al. [5], the prevalence of obesity among adult Nigerians ranged from 8.1 to 22.2%. The prevalence reported in this study is high. An explosion in the rates of obesity and high blood pressure has been reported with a concomitant rise in deaths from cardiovascular disease, diabetes, cancer and chronic respiratory disease which has become faster in Africa than anywhere else in the world [4]. Choi et al. [20] reported that compared with adults who were not obese, those with abdominal obesity without general obesity and both general and abdominal obesity had elevated risk of major adverse cardiac events while those with general obesity without abdominal obesity did not, suggesting that individuals should guard against abdominal obesity particularly.

General obesity alone and abdominal obesity alone were more likely among those aged 41-60 years. Significance was observed with general obesity alone. Participants within 41-60 years were significantly less likely to have combined obesity than their younger counterparts. Other researchers [21, 22] have also reported significant association between age and obesity. Khabazkhoob et al. [23] studied prevalence of overweight and obesity in middle-age population and reported that people over the age of 54 years were more likely to have BMI ≥ 25 kg/m^2^ than those within 40–44 years. Naturally, weight increases with age and with advancement in age, conscious vigorous physical activity decreases leading to weight gain and retention of weight gained over a period of time hence the higher likelihood of general obesity alone observed among the older adults was not a surprise. Childhood obesity has been linked with adulthood obesity implying that weight gained in earlier years is not easily lost.

Females were significantly at reduced risk of abdominal obesity alone and combined obesity but almost 8 times at higher risk of general obesity alone. Zubery et al. [22] also reported significant association between gender and obesity with females at higher risk than males. This result was attributed to lower energy expenditure among females. In a study to determine gender differences in energy expenditure during a 10-minute walking exercise with backpack and double-pack loads, Li et al. [24] reported that healthy young female participants carried a heavy double-pack with less energy cost compared with their male counterparts. Multiparous females may be at higher risk because of the difficulty associated with postnatal loss of weight gained during pregnancy.

Never married was associated with increased likelihood of abdominal obesity alone and combined obesity but not general obesity alone. Significance observed with combined obesity requires further investigation as it contradicts existing literature. The lower risk of general obesity alone among the never married agrees with the findings of other researchers [21, 22] who reported significant positive association of marital status with obesity with higher risk among married persons. In a study [25] that examined body weight, marital status and changes in marital status, living without a partner, either being divorced or never married, was associated with lower body weight but cohabiters and married respondents weighed more. These findings imply that married persons are predisposed to obesity probably due to the stability offered by married life and consequences of pregnancy weight gain. General obesity alone and combined obesity were more likely among those with any form of formal education; significance was observed with general obesity alone. This is in line with the report of Simo et al. [26] but contrary to the report of lower risk of obesity among educated women [21]. Our finding may be attributed to consumption of energy dense foods which according to Simo et al. [26] may be a function of improved economic status associated with higher education. That education improves literacy and understanding, promotes better life style choices and helps the development of good health habits depends on the ability of the individual to live by what has been learnt.

We observed that those who did not skip meals had lower risk of abdominal obesity alone and combined obesity. Only the association with abdominal obesity alone attained significance. This was expected but those who did not skip meals being at higher risk of general obesity was a surprise and may be attributed to time of meal intake, quantity and quality of what was consumed. We also observed that consumption of 3 or more meals daily was significantly associated with greater risk of abdominal obesity alone. Consumption of energy dense foods coupled with low/moderate physical activity has propensity of leading to weight gain and its retention. Despres [27] stated that the risk of abdominal obesity increased with low physical activity and high energy diets. Lopez-Minguez et al. [28] showed that late consumption of breakfast, lunch and supper was associated with less weight loss than early meal consumption.

Participants who reported vigorous physical activity were unlikely to have abdominal obesity alone and general obesity alone. This is in line with the observations of other researchers [29, 30]. Wanner et al. [29] reported significant association of physical inactivity with overweight/obesity both cross sectionally and longitudinally among 18 – 60 year-olds in eight regions in Switzerland. Silva et al. [30] reported that adiposity was lower among those who were consistently active while Lee et al. [31] found no significant association between self-reported total physical activity with body mass index and waist circumference measurement. Through physical activity, energy expenditure is increased with possibility of healthy energy balance and weight status. The association reported in this study with combined obesity may be a function of inconsistent vigorous activity.

### Strengths and limitations

The strength of this study lies in the use of multistage (probability) sampling technique and the fact that it is a community-based study. However, the cross-sectional design used in this study did not assess cause–effect relationships. The age group studied did not extend to the elderly and the sample size was relatively small making generalization of the research outcome to the entire adults in Enugu State, Southeast Nigeria inappropriate. There is possibility of recall bias with self-reported variables. Besides, energy intake of the respondents was not studied.

## Conclusion

Obesity is a severe public health problem among adults in rural Nigerian communities. Age, sex, skipping meals, ≥3 daily meal intake, having any form of formal education, never married and self-reported vigorous physical activity were identified significant predictors. The positive association between combined obesity and never married requires further investigation. These factors though not new, should continue to be the focus of nutrition and health education as interventions for effective prevention and control of obesity among Nigerian adults in line with WHO [4] global target to halt the rise in obesity.

## Data Availability

Data generated from this study on which the results are based are available from the corresponding author on reasonable request.

## Abbreviations

CVD: Cardiovascular diseases
NCD: Non communicable diseases
BMI: Body mass index
WC: Waist circumference
WHO: World Health Organization
COR: Crude odds ratio
AOR: Adjusted odds ratio
C.I.: Confidence interval
